# Infections among individuals with multiple sclerosis, Alzheimer’s disease and Parkinson’s disease

**DOI:** 10.1093/braincomms/fcad065

**Published:** 2023-03-16

**Authors:** Yihan Hu, Kejia Hu, Huan Song, Yudi Pawitan, Fredrik Piehl, Fang Fang

**Affiliations:** Unit of Integrative Epidemiology, Institute of Environmental Medicine, Karolinska Institutet, Stockholm, Sweden; Unit of Integrative Epidemiology, Institute of Environmental Medicine, Karolinska Institutet, Stockholm, Sweden; West China Biomedical Big Data Center, West China Hospital, Sichuan University, Chengdu, China; Med-X Center for Informatics, Sichuan University, Chengdu, China; Centre of Public Health Sciences, Faculty of Medicine, University of Iceland, Reykjavik, Iceland; Department of Medical Epidemiology and Biostatistics, Karolinska Institutet, Stockholm, Sweden; Department of Clinical Neuroscience, Karolinska Institutet, Stockholm, Sweden; Unit of Integrative Epidemiology, Institute of Environmental Medicine, Karolinska Institutet, Stockholm, Sweden

**Keywords:** multiple sclerosis, Alzheimer’s disease, Parkinson’s disease, infections

## Abstract

A link between neurodegenerative diseases and infections has been previously reported. However, it is not clear to what extent such link is caused by confounding factors or to what extent it is intimately connected with the underlying conditions. Further, studies on the impact of infections on mortality risk following neurodegenerative diseases are rare. We analysed two data sets with different characteristics: (i) a community-based cohort from the UK Biobank with 2023 patients with multiple sclerosis, 2200 patients with Alzheimer’s disease, 3050 patients with Parkinson’s disease diagnosed before 1 March 2020 and 5 controls per case who were randomly selected and individually matched to the case; (ii) a Swedish Twin Registry cohort with 230 patients with multiple sclerosis, 885 patients with Alzheimer’s disease and 626 patients with Parkinson’s disease diagnosed before 31 December 2016 and their disease-free co-twins. The relative risk of infections after a diagnosis of neurodegenerative disease was estimated using stratified Cox models, with adjustment for differences in baseline characteristics. Causal mediation analyses of survival outcomes based on Cox models were performed to assess the impact of infections on mortality. Compared with matched controls or unaffected co-twins, we observed an elevated infection risk after diagnosis of neurodegenerative diseases, with a fully adjusted hazard ratio (95% confidence interval) of 2.45 (2.24–2.69) for multiple sclerosis, 5.06 (4.58–5.59) for Alzheimer’s disease and 3.72 (3.44–4.01) for Parkinson’s disease in the UK Biobank cohort, and 1.78 (1.21–2.62) for multiple sclerosis, 1.50 (1.19–1.88) for Alzheimer’s disease and 2.30 (1.79–2.95) for Parkinson’s disease in the twin cohort. Similar risk increases were observed when we analysed infections during the 5 years before diagnosis of the respective disease. Occurrence of infections after diagnosis had, however, relatively little impact on mortality, as mediation of infections on mortality (95% confidence interval) was estimated as 31.89% (26.83–37.11%) for multiple sclerosis, 13.38% (11.49–15.29%) for Alzheimer’s disease and 18.85% (16.95–20.97%) for Parkinson’s disease in the UK Biobank cohort, whereas it was 6.56% (−3.59 to 16.88%) for multiple sclerosis, −2.21% (−0.21 to 4.65%) for Parkinson’s disease and −3.89% (−7.27 to −0.51%) for Alzheimer’s disease in the twin cohort. Individuals with studied neurodegenerative diseases display an increased risk of infections independently of genetic and familial environment factors. A similar magnitude of risk increase is present prior to confirmed diagnosis, which may indicate a modulating effect of the studied neurological conditions on immune defences.

## Teaser

Hu *et al*. reported that individuals with multiple sclerosis, Alzheimer’s disease or Parkinson’s disease were more likely to experience hospital-treated infections and increased mortality, compared with others, and that infections contributed little to the increased mortality.

## Graphical Abstract

**Graphical abstract:**
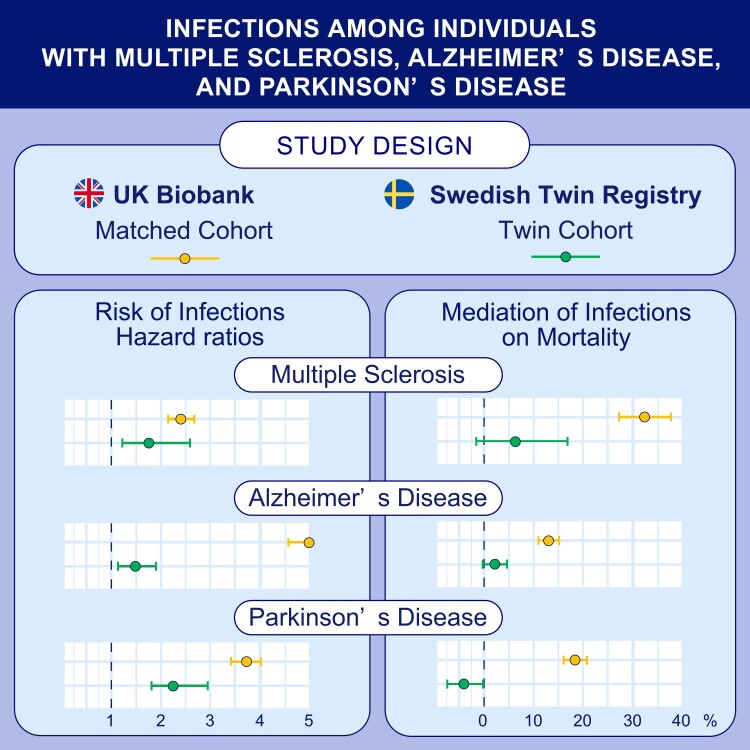

## Introduction

Neurodegenerative diseases are a group of disorders characterized by a progressive loss of neuronal structure or function.^1^ An estimated 50 million people worldwide suffer from dementia or Alzheimer’s disease (AD).^2^ The corresponding numbers are 7–10 million for Parkinson’s disease (PD) and more than 2 million for multiple sclerosis (MS).^3,4^ AD is the most common neurodegenerative disease and is characterized by memory impairment and problems in daily living tasks.^5^ With the early death of dopaminergic neurons in substantia nigra, patients with PD experience symptoms like rest tremor, rigidity, bradykinesia, postural and gait impairment.^6^ Although not conventionally considered a neurodegenerative disease, earlier inflammatory phases of MS are characterized by progressive damage to the central nervous system, eventually leading to considerable disability among those affected.^7,8^

A link between infections and neurodegenerative diseases has been previously reported, with the triggering of neuroinflammatory processes as a potential causative mechanism.^9,10^ Accordingly, previous studies have mainly considered infections as a risk factor for neurodegenerative diseases.^11-16^ For instance, Sipilä *et al*.^16^ reported an association between infections and a higher risk of dementia, especially AD and vascular dementia. The risk of infections among patients with an established neurodegenerative disease has, however, been less systematically explored. Several challenges exist for disentangling the relation between neurodegenerative diseases and infections, as they share common risk factors, most importantly ageing,^17^ which contribute to altered immunity both systemically and in organ-specific locations, such as the central nervous system and respiratory organs.^18,19^ Additionally, behavioural changes, such as a more sedentary lifestyle, may also contribute to a higher risk of infections among those affected by neurodegenerative diseases.^20,21^ It, therefore, remains unclear to what extent a higher-than-expected comorbidity between neurodegenerative diseases and infections is caused by confounding factors.

Patients with neurodegenerative diseases display higher-than-expected mortality,^22-24^ which is not surprising given the progressive nature of these conditions. At the same time, infections have been proposed to play a role in the progression of neurodegenerative diseases. For example, Buljevac *et al*.^25^ reported that infections can trigger relapses and worsening of disability among patients with MS. Systematic infections were found to amplify brain cytokine and accelerate the disease progression of AD.^26,27^ Recently, coronavirus disease 2019 (COVID-19) was observed to worsen PD progression.^28^ However, these findings are often based on relatively small-sized materials and are not suitable for determining to what extent infections explain the increased mortality observed in neurodegenerative diseases.

In order to address these questions, we conducted a comprehensive analysis to examine whether three neurodegenerative diseases (i.e. MS, AD and PD) are associated with the risk of infections. We used two complementary cohorts, namely, a community-based matched cohort within the UK Biobank and a twin cohort within the Swedish Twin Registry (STR). The latter was used to control for potential confounding due to shared genetic and non-genetic factors within a twin pair, which has been rarely done in previous studies. To limit the impact of delay in the ascertainment of neurodegenerative diseases, we also calculated the risk of infections during the 5-year period before the respective neurodegenerative disease was clinically confirmed. To explore the potential role of infections on neurodegenerative disease progression, we further assessed the role of infections on the risk of mortality among patients diagnosed with MS, AD and PD. We hypothesized that patients with MS, AD and PD are at a higher risk of infections and that infections partly mediate the risk of mortality among these patients.

## Materials and methods

### Data sources

The UK Biobank is a large-scale prospective cohort study comprising >500 000 participants aged 40–69 years who were recruited between 2006 and 2010.^29^ Participants were assigned to 1 of 22 assessment centres across England, Scotland and Wales, and provided self-reported information on sociodemographic characteristics, lifestyle, etc. at enrolment. Hospital inpatient and mortality data from multiple national data sets are periodically linked to the data base. Detailed study design and specified data collection procedures of the UK Biobank have been described previously.^29^ UK Biobank ethical approval was received from the NHS Research Ethics Committee (REC reference: 16/NW/0274). The present study was approved under the UKB application 76517 and by the Swedish Ethical Review Authority (DNR: 2022-01516-01).

The STR was started in the late 1950s and contains information on twins born in Sweden since 1886.^30^ Questionnaire data were additionally derived from substudies of STR, including Q61, Q63, Q67, Q70 (twins born in 1886–1925), Q73 (twins born in 1926–58), SALT (twins born in 1944–58), STAGE (twins born in 1959–85), YATSS (twins born in 1985–92) and CATSS (twins born 1992 onward). Data from STR were additionally cross-linked to the Swedish National Patient and Cause of Death Registers using the Swedish unique personal identification numbers. The Patient Register includes nationwide data on inpatient care since 1987 (>80% during 1981–85) and outpatient hospital visits since 2001.^31^ Different Swedish revisions of the International Classification of Diseases (ICD) codes were used to identify different diagnoses in the register (ICD-8 in 1969–86, ICD-9 in 1987–96 and ICD-10 since 1997). Informed consent was provided by all participants of the STR. The present study was approved by the Swedish Ethical Review Authority (DNR: 2021-02994).

### Study design

Initially, we conducted a matched cohort study using the UK Biobank data set. In the exposed group, we included 2023 patients with MS, 2200 patients with AD and 3050 patients with PD with a diagnosis of the respective disease before 1 March 2020 on the basis of UK Biobank inpatient hospital data, with date of first hospital visit concerning the disease (either primary or secondary diagnosis) used as index date. In the unexposed group, we randomly selected five individuals among the participants of UK Biobank, who were individually matched to the respective patient with MS/AD/PD by age, sex and Charlson comorbidity index (CCI), using the method of incidence density sampling.^32^ To be eligible, matched controls had to be alive and free of the respective MS/AD/PD diagnosis of the matched patient on the date of selection. As a proxy for general health status,^33^ we calculated CCI (0, 1 or ≥2) for each participant at the date of diagnosis or selection, using diagnoses in the inpatient hospital data (Supplementary Table 1).^34^ Dementia was excluded from this calculation. Individuals with more than one MS/AD/PD diagnosis could independently be included in more than one analysis (*n* = 182 individuals).

We subsequently performed a twin cohort study using the STR, in order to control for potential unknown and unmeasured confounding due to shared genetic and non-genetic factors within a twin pair. In this analysis, we compared the risk of infections among patients with MS/AD/PD to their unaffected co-twins. We selected all complete twin pairs in the STR, for which one twin had been diagnosed with MS/AD/PD before 31 December 2016, and the un-affected co-twin was still alive at the index date. We identified in this analysis 230 twin pairs of MS, 885 twin pairs of AD and 626 twin pairs of PD.

In both cohorts, MS was ascertained by ICD-9 code 340 and ICD-10 code G35. AD was ascertained by ICD-9 code 331.0 (also 290.0 and 290.1 for the primary diagnosis) and ICD-10 codes F00 and G30. PD was ascertained by ICD-9 code 332.0 and ICD-10 code G20.

### Infections

In the UK Biobank, we identified all inpatient care episodes for infections using inpatient hospital data, whereas in STR, we identified all inpatient care episodes for infections before 2001, and both in- and outpatient care infections since 2001 through the Swedish National Patient Register. Both primary and secondary diagnoses were used as ascertainment of infections. We further categorized episodes of infections by site (i.e. genitourinary, respiratory and other/unspecified) and type (i.e. bacterial, viral and other), and additionally studied pneumonia as a separate entity. ICD codes used to identify infections are listed in Supplementary Table 2. If an individual had more than one infection diagnosis during an episode of hospital admission, we used the date of the first infection in the analysis of subtypes of infections.

### Follow-up of the cohorts

In both the UK Biobank and STR cohorts, we defined date of diagnosis as the index date for the patient and their matched unexposed individuals and unaffected co-twin, respectively. We then followed exposed and un-exposed individuals from the index date until the first diagnosis of infection, death, diagnosis of MS/AD/PD (unexposed groups only) or end of follow-up (1 March 2020 and 31 December 2016 in the UK Biobank and STR cohorts, respectively), whichever came first. Accordingly, we identified mortality of participants before 1 March 2020 in the UK Biobank, and mortality of participants before 31 December 2016 in STR, according to the Swedish Causes of Death Register.

### Statistical analysis

In main analysis, we used hazard ratios (HRs) with 95% confidence intervals (CIs), derived from Cox models, to estimate the risk of infections in relation to MS, AD or PD. Time since index date was used as the underlying timescale. In the UK Biobank cohort, we stratified all analyses by matching variables (i.e. age, sex and CCI) and additionally adjusted for Townsend deprivation score (as continuous variable), educational attainment (college degree, A-level, O-level, Certificate of Secondary Education or equivalent, National Vocation Qualifications or equivalent, other professional qualifications or unknown) and annual household income (<£18 000, £18 000–30 999, £31 000–51 999, £52 000–100 000, >£100 000 or unknown).

In the STR cohort, we stratified the analyses by twin pair and adjusted for sex and educational attainment (compulsory school or elementary school, upper secondary education, university, others or unknown). In both cohorts, we performed analyses first for any infection and then for different sites and types of infections. Only first occurrence of any or a specific site/type of infection was considered in the analyses. For example, with an individual being hospitalized for a bacterial pneumonia on 28 February 2000 and influenza on 20 January 2002, the first date was considered for any, bacterial and respiratory tract infection, whereas the latter date was considered for viral infection.

Assuming the effect of infections on mortality is causal, we performed causal mediation analyses of survival outcomes based on Cox models. Details of this method have been described previously.^35^ In brief, the mediation proportion by infections was calculated by comparing indirect effect through infections (whether infected during follow-up) and total effect of MS/AD/PD on mortality. Assumptions used in this method have also been described previously.^35^ Briefly, apart from measured confounders, we assumed that there was no confounding in: (i) the association of MS/AD/PD with risk of mortality; (ii) the association of infections with risk of mortality, conditioned on MS/AD/PD; (iii) the association of MS/AD/PD with the risk of infections; and (iv) the association of infections with risk of mortality caused by MS/AD/PD.

The mediation analyses in the UK Biobank cohort were conditioned on demographic characteristics and baseline comorbidities, with further adjustment for socioeconomic status. In the STR cohort, with the advantages of the twin design, the mediation analyses were inherently controlled for the genetic background and other familial factors shared between twins. We used the Wald test to test the statistical significance of the different estimates between groups and used *Z*-test to compare the HRs between the UK Biobank cohort and the STR cohort.^36^

Finally, given the ascertainment methods used in the UK Biobank and STR, it is likely that there was a detection delay between the index date of the studied neurodegenerative diseases and actual date of diagnosis, especially if the index date was ascertained through the inpatient care setting. We therefore also compared the odds of experiencing at least one infection during the 5 years before the index date of the patients with MS/AD/PD and their matched controls or unaffected co-twins, to allow a 5-year delay of detection. For this purpose, we used odds ratios with 95% CIs derived from conditional logistic regression to compare the prevalence of infections during the 5 years before index date between patients with MS/AD/PD and their matched controls as well as unaffected co-twins.

All analyses were conducted by Python software (version 3.8) and R software (version 4.1). A two-sided *P* < 0.05 was considered statistically significant.

## Results

Characteristics of participants in the UK Biobank cohort and the STR cohort are shown in Table 1 and Supplementary Table 3. In the UK Biobank cohort, we included 2023 MS patients with 10 115 matched controls (mean age 54.7 years, 28.0% men), 2200 patients with AD with 10 999 matched controls (mean age 73.2 years, 48.5% men) and 3050 patients with PD with 15 249 matched controls (mean age 69.1 years, 61.5% men). In the STR cohort, we included 230 patients with MS (mean age 47.9 years, 26.5% men) and 230 unaffected co-twins (38.3% men), 885 patients with AD (mean age 77.2 years, 34.6% men) and 899 unaffected co-twins (34.6% men, including 14 triplets), and 626 patients with PD (mean age 71.5 years, 51.3% men) and 626 unaffected co-twins (50.2% men). Patients with neurodegenerative diseases were older and more likely to be men in the UK Biobank, compared with the STR cohort.

**Table 1:** Characteristics of individuals included in the cohort studies of MS, AD and PD, based on the UK biobank and the STR

	UK Biobank						Swedish Twin Registry					
Characteristics	MS patients (*n* = 2023)	Matched controls (*n* = 10 115)	AD patients (*n* = 2200)	Matched controls (*n* = 10 999)	PD patients (*n* = 3050)	Matched controls (*n* = 15 249)	MS patients (*n* = 230)	Co-twin controls (*n* = 230)	AD patients (*n* = 885)	Co-twin controls (*n* = 899)	PD patients (*n* = 626)	Co-twin controls (*n* = 626)
Number of controls that developed MS/AD/PD during follow-up, *n* (%)		19 (0.2)		75 (0.7)		83 (0.5)		8 (3.4)		112 (12.5)		13 (2.1)
Age, mean (SD)	54.7 (10.1)	54.7 (10.1)	73.2 (5.1)	73.2 (5.1)	69.1 (7.2)	69.1 (7.2)	47.9 (15.0)	47.9 (15.0)	77.2 (7.2)	77.2 (7.2)	71.5 (10.0)	71.5 (10.0)
Sex, *n* (%)												
Male	567 (28.0)	2835 (28.0)	1067 (48.5)	5335 (48.5)	1877 (61.5)	9385 (61.5)	61 (26.5)	88 (38.3)	306 (34.6)	336 (37.4)	321 (51.3)	314 (50.2)
Female	1456 (72.0)	7280 (72.0)	1133 (51.5)	5664 (51.5)	1173 (38.5)	5864 (38.5)	169 (73.5)	142 (61.7)	579 (65.4)	563 (62.6)	305 (48.7)	312 (49.8)

As expected, more unaffected co-twins of the STR cohort developed the corresponding neurodegenerative disease of the affected twin during follow-up, compared with the matched controls of the UK Biobank cohort (3.4 versus 0.2% in MS, 12.5 versus 0.7% in AD and 2.1 versus 0.5% in PD). In the UK Biobank cohort, we noted little difference in Townsend deprivation index and educational attainment between exposed and unexposed groups of MS and PD, while patients with AD were more likely to have higher Townsend deprivation index and lower educational attainment than their matched controls. We also observed lower annual household income among patients with MS, AD or PD (<£18 000: 25.5 versus 19.8% in MS, 33.9 versus 29.1% in AD and 29.1 versus 25.9% in PD) compared with the matched controls.

**Figure 1:**
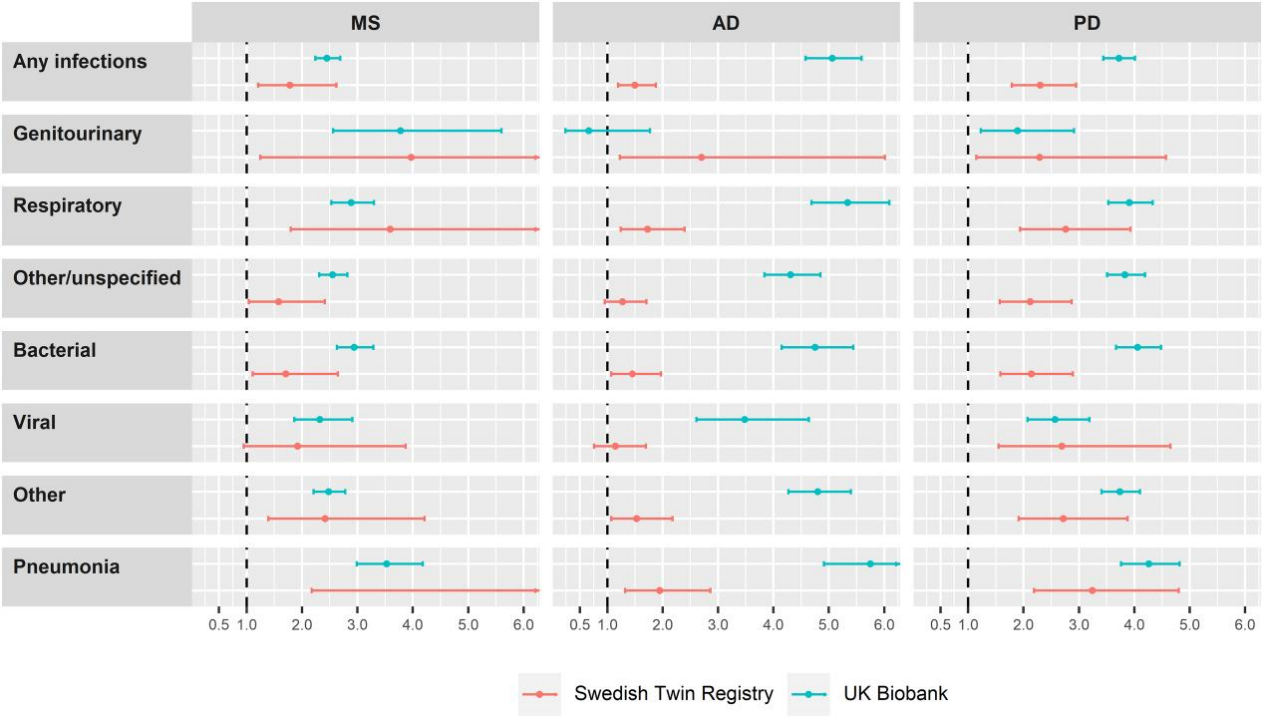
**Associations of MS, AD and PD with the risk of infections.** HRs and 95% CIs are derived from Cox models. Models in the UK Biobank cohort are conditioned on matching factors (age, sex and CCI) and additionally adjusted for annual household income, educational attainment and the Townsend deprivation index (MS patients *n* = 2023, matched controls *n* = 10 115; AD patients *n* = 2200, matched controls *n* = 10 999; PD patients *n* = 3050, matched controls *n* = 15 249). Models in the STR cohort are conditioned on twin pair and additionally adjusted for sex and educational attainment (MS patients *n* = 230, co-twin controls *n* = 230; AD patients *n* = 885, co-twin controls *n* = 889; PD patients *n* = 626, co-twin controls *n* = 626).

In the UK Biobank cohort, the crude incidence rate of any infection was 40.84 and 16.69 per 1000 person-years for patients with MS and their matched controls, 222.69 and 45.70 per 1000 person-years for patients with AD and their matched controls, and 110.94 and 32.69 per 1000 person-years for patients with PD and their matched controls (Supplementary Table 4). We observed an association between neurodegenerative disease and risk of infections, with a fully adjusted HR of 2.45 (95% CI 2.24–2.69) for MS, 5.06 (95% CI 4.58–5.59) for AD and 3.72 (95% CI 3.44–4.01) for PD (Fig. 1).

In the STR cohort, we observed 107 and 73 cases of infections among twins with MS and their unaffected co-twins, respectively, while the corresponding numbers were 240 and 286 for AD and 266 and 208 for PD, respectively (Supplementary Table 5). The fully adjusted HR was 1.78 (95% CI 1.21–2.62) for MS, 1.50 (95% CI 1.19–1.88) for AD and 2.30 (95% CI 1.79–2.95) for PD, respectively (Fig. 1). In the UK Biobank cohort, we observed a positive association between neurodegenerative disease and different sites of infections, except for genitourinary infections in AD (HR 0.66; 95% CI 0.24–1.77; Fig. 1). In the analyses by site or type of infections, we found close estimates between subgroups. In the STR cohort, we also found a positive association for most types of infections, except for AD and viral infections (HR 1.14; 95% CI 0.76–1.70; Fig. 1).

Patients with neurodegenerative diseases displayed increased mortality compared with their matched controls or unaffected co-twins (Table 2). In the UK Biobank cohort, HR was 2.67 (95% CI 2.27–3.14) for MS, 8.74 (95% CI 7.75–9.86) for AD and 5.20 (95% CI 4.72–5.72) for PD. In the STR cohort, HR was 4.25 (95% CI 2.05–8.83) for MS, 2.89 (95% CI 2.40–3.49) for AD and 3.88 (95% CI 3.05–4.94) for PD. We observed greater risk increase of mortality with AD and PD in the STR cohort compared with the UK Biobank cohort (*P* for difference < 0.001 for AD and *P* for difference = 0.03 for PD).

**Table 2:** Associations of multiple sclerosis (MS), Alzheimer’s disease (AD) and Parkinson’s disease with the risk of mortality and mediation proportion of infections in these associations

				HR (95% CI)		Mediation Proportion (95% CI)
	All-cause mortality	Patients with MS/AD/PD (cases/person-years, IR per 1000 person-years)	Matched references/co-twin references (cases/person-years, IR per 1000 person-years)	Total effect	Direct effect	
UK Biobank^a^	MS	243/23 304 (10.43)	489/119 595 (4.09)	2.67 (2.27–3.14)	1.94 (1.63–2.31)	31.89% (26.83–37.11%)
	AD	785/5004 (156.87)	621/33 383 (18.60)	8.74 (7.75–9.86)	7.03 (6.20–7.98)	13.38% (11.49–15.29%)
	PD	892/14 190 (62.86)	1049/80 887 (12.97)	5.20 (4.72–5.72)	3.87 (3.49–4.30)	18.85% (16.75–20.97%)
Swedish Twin Registry^b^	MS	58/2684 (21.61)	27/3013 (8.96)	4.25 (2.05–8.83)	4.48 (2.08–9.65)	6.56% (−3.59 to 16.88%)
	AD	610/3820 (159.69)	388/5511 (70.40)	2.89 (2.40–3.49)	2.83 (2.35–3.42)	2.21% (−0.21–4.65%)
	PD	398/3813 (104.38)	271/5472 (49.52)	3.88 (3.05–4.94)	4.29 (3.30–5.57)	−3.89% (−7.27 to −0.51%)

a Conditioned on pair matched with age, sex and Charlson comorbidity index, and additionally adjusted for annual household income, educational attainment and Townsend deprivation index. ^b^Conditioned on twin pair and additionally adjusted for sex and education attainment.

Adjusting for infections during follow-up did not change the HRs greatly. In the UK Biobank cohort, the mediation proportion by infections was estimated at 31.89% (95% CI 26.83–37.11%) in MS, 13.38% (95% CI 11.49–15.29%) in AD and 18.85% (95% CI 16.75–20.97%) in PD. In the STR, no mediation proportion by infections was discerned in MS (6.56%; 95% CI −3.59 to 16.88%), AD (2.21%; 95% CI −0.21 to 4.65%) and PD (−3.89%; 95% CI −7.27 to −0.51%; *P* = 0.05).

**Figure 2:**
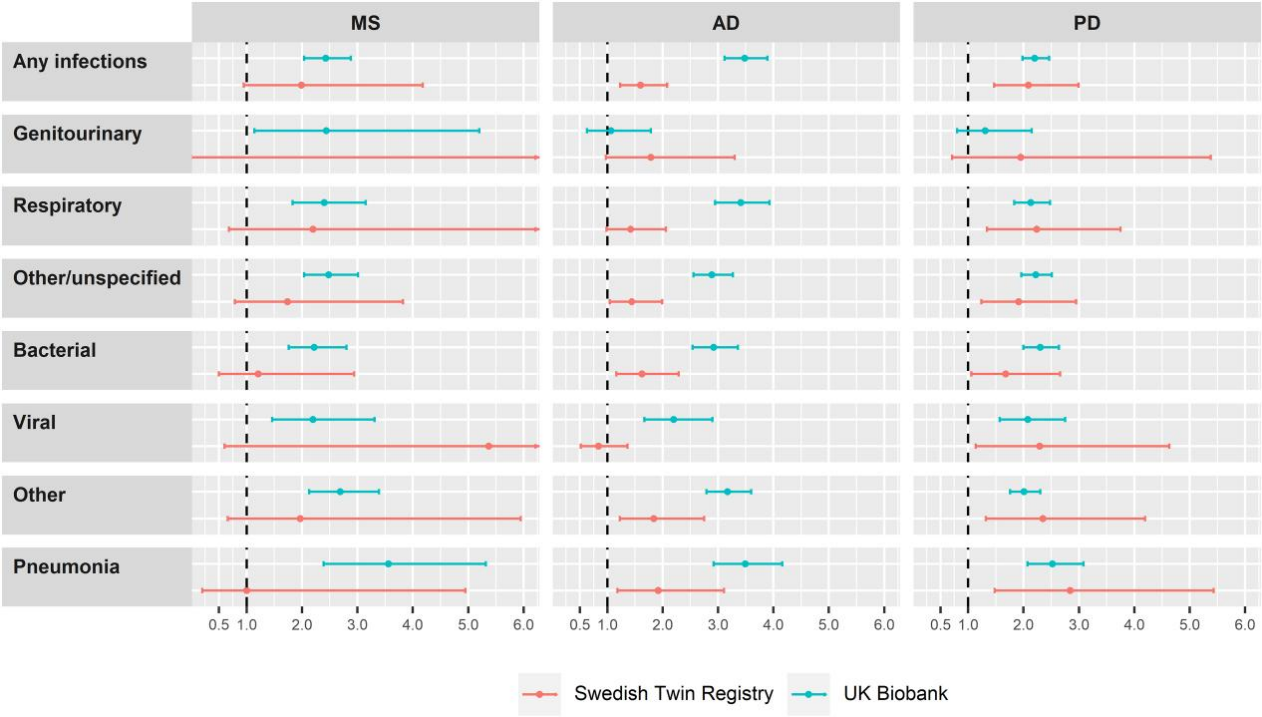
**Associations of MS, AD and PD with the risk of infections during the 5 years before diagnosis.** ORs and 95% CIs are derived from conditional logistic regression models. Models in the UK Biobank cohort are conditioned on matching factors (age, sex and CCI), and additionally adjusted for annual household income, educational attainment and Townsend deprivation index (MS patients *n* = 2023, matched controls *n* = 10 115; AD patients *n* = 2200, matched controls *n* = 10 999; PD patients *n* = 3050, matched controls *n* = 15 249). Models in the STR cohort are conditioned on twin pair and additionally adjusted for sex and educational attainment (MS patients *n* = 230, co-twin controls *n* = 230; AD patients *n* = 885, co-twin controls *n* = 889; PD patients *n* = 626, co-twin controls *n* = 626).

In the analyses of infections within 5 years before index date, we also found a higher prevalence of any infection and most types and sites of infection among patients with MS/AD/PD, compared with their matched controls or unaffected co-twins, although there was only a trend for MS in the STR cohort (Fig. 2, Supplementary Tables 5 and 7).

## Discussion

In the present study, based on the analyses of a matched community-based cohort and a twin cohort, we found that individuals with MS, AD or PD displayed an elevated risk of infections. The risk increase remained robust after adjusting for potential confounders, including familial confounders due to shared genetic and non-genetic factors within a twin pair. In the subgroup analyses, we found positive associations for most of types and sites of infections, suggesting that the risk increase of infections was likely generic rather than specific to certain pathogens or sites. Notably, we observed a similar magnitude of risk increase and pattern of infections within 5 years before index date, indicating that this effect is present earlier in the disease course. We also found that patients with MS, AD or PD had an increased risk of mortality compared with unrelated population controls and their unaffected co-twins. In the community-based cohort, we found that infections mediated some of the increased mortality, while no mediating effect was evident in the twin cohort.

An increased risk of infections among patients with MS with different disease-modifying therapies (DMTs) affecting the normal physiological functions of the immune defence has been discussed in previous studies.^37,38^ It is, however, less clear if MS itself contributes to an increased risk of infections.^39^ To our best knowledge, the present study is the first to assess the risk of infections longitudinally among patients with MS, including the 5 years preceding ascertainment of the diagnosis, although a previous Swedish study observed increased incidence of infections in the year prior to diagnosis as well as in the following years after diagnosis of MS.^40^ The consistently increased risk of infections in the pre- and post-diagnostic period of MS can therefore not be explained by DMTs alone, also taking into account that the penetration of more potently acting DMTs was limited in Sweden for much of the study period.^41^ A stronger association was noted between MS and infections in the UK Biobank cohort, compared with the STR cohort. A potential explanation for the difference is shared risk factors of MS and infections, which could be better adjusted for in the STR cohort. Another potential explanation is the fact that we ascertained MS cases from only inpatient care data in the UK Biobank, whereas both inpatient and outpatient care data was available in the STR. The UK Biobank patients with MS might therefore have more severe forms of MS compared with the patients with STR MS, thus amplifying differences. In addition, the age at inclusion was higher in the UK Biobank cohort, potentially due to greater ascertainment delay. Interestingly, however, high point estimates for an association with MS were noted for pneumonia and respiratory tract infections in the 5 years before diagnosis in both data sets. Increasing experimental evidence has indeed pointed towards a link between lung microbiota and triggering of neuroinflammation,^42,43^ where a positive association between pneumonia and subsequent diagnosis of MS has been observed also in clinical setting.^44^

Positive associations of AD and PD with risk of infections have also been noted previously. For instance, aspiration pneumonia is reported to be a common cause of death in patients with AD,^45-48^ while patients with PD are at a higher risk of genitourinary and bacterial infections.^49,50^ An increased risk of severe COVID-19 has also been reported in both AD and PD.^34,51^ It is however less clear whether patients with AD or PD, especially in earlier disease phases, are more prone to infections in general or to a specific type or site of infection. The fact that patients with both PD and AD displayed increased risks of infections in general, including bacterial and viral infections, and at different sites, even in the years before the disease was clinically ascertained therefore represents a novel finding. As in MS, a mechanistic link has mostly focused on the potential role of infections as a triggering or facilitating factor for neurodegenerative conditions.^52-55^ However, it cannot be excluded that a proportion of a potential causality has the opposite direction. For instance, experimental evidence shows that ischaemic brain processes also affect systemic immunity,^56-58^ which is also supported by clinical observations.^59^ Since vagal reflex circuits have been shown to potently affect T-cell responses in inflammation and infection,^60^ it therefore remains a possibility that such effects may encompass a wider range of neurological conditions, including neurodegenerative diseases.

Patients with MS, AD or PD display increased mortality,^22-24^ and infections have been suggested to play a role in disease exacerbation.^18,61^ However, to what extent increased risk of mortality can be attributed to infections remains less clear. In the two data sets used here, we found significantly increased risk of mortality across all three diseases. These observations corroborate results of previous studies,^24,62-64^ but add an additional level of support by the replication in the twin setting. In the UK Biobank cohort, we found that infections explained part of the increased risk of mortality, especially among patients with MS. In contrast, in the twin data set, no mediating effect was observed, indicating confounding by genetic or familial environment factors in the mediation effects. Future studies are however needed to validate these findings, and if verified, examine the potential factors explaining the recorded discrepancies.

A strength with the present study is the combined use of the UK Biobank and the STR to perform both a community-based matched cohort study and a twin study. The rich information in the UK Biobank enabled us to adjust for a wide range of potential confounders, while the within-twin pair analysis provided a unique opportunity to assess the role of familial confounding. This study, however, also suffers from certain limitations. The first concern is the lack of data on the clinical characteristics and treatments of MS/AD/PD, precluding the possibility of studying the roles of potential modifying factors, such as disability level or immune-modulating treatments, for determining the association of these diseases with infections. We also lacked information on potentially important confounders such as smoking and body mass index. A second concern is that we could identify only a diagnosis of neurodegenerative diseases through inpatient care data in the UK Biobank cohort, and that, while also outpatient specialized care data were available for the STR cohort, they were not for the entire study period. In order to limit the impact of delayed ascertainment of these diagnoses, we also analysed the 5 years before ascertainment of MS/AD/PD, finding associations of similar magnitude. Still, it cannot be excluded that disease was diagnosed prior, at least in a proportion of patients. Finally, our definition included infections recorded only from hospital visits, therefore restricting the interpretation to more severe forms of infections. It is also possible that patients in advanced stages of neurodegenerative diseases are cared for in nursing homes, where treatments for infections might be administered locally by a general practitioner and therefore not captured. In particular, this may affect the association between infections and mortality in patients with late-stage disease.

## Conclusion

Using both a large community-based cohort with matched controls and a twin cohort, we identified an increased risk of both infections and mortality among patients with MS, AD and PD independent of genetic and familial environmental factors. An increased risk of infections was also evident in the 5 years preceding the index date of the neurodegenerative diseases, which may depend on the fact that infections can act as triggers for neurodegenerative diseases or that such diseases affect systemic immunity, or both. However, although infections mediated some of the increased mortality seen among patients with MS/PD/AD in the community-based cohort, the association largely disappeared in the within-twin pair analysis.

## Supplementary Material

fcad065_Supplementary_Data

## Data Availability

Data from the UK Biobank (http://www.ukbiobank.ac.uk/) are available to all researchers upon application. Part of this research was conducted using the UK Biobank Resource under Application 76517. Data from STR are available with permission from the STR.

## Abbreviations

AD =: Alzheimer’s disease
CCI =: Charlson comorbidity index
CI =: confidence interval
DMT =: disease-modifying therapy
HR =: hazard ratio
ICD =: International Classification of Diseases
MS =: multiple sclerosis
PD =: Parkinson’s disease
STR =: Swedish Twin Registry

## References

[1] Dugger BN , Dickson DW. Pathology of neurodegenerative diseases. Cold Spring Harb Perspect Biol. 2017;9(7):a028035. 10.1101/cshperspect.a028035 PMC5495060 28062563

[2] Scheltens P , Strooper BD, Kivipelto M, et al Alzheimer’s disease. Lancet. 2021;397(10284):1577–1590. 33667416 10.1016/S0140-6736(20)32205-4 PMC8354300

[3] Tysnes OB , Storstein A. Epidemiology of Parkinson’s disease. J Neural Transm. 2017;124(8):901–905. 28150045 10.1007/s00702-017-1686-y

[4] Reich DS , Lucchinetti CF, Calabresi PA. Multiple sclerosis. N Engl J Med. 2018;378(2):169–180. 29320652 10.1056/NEJMra1401483 PMC6942519

[5] Burns A , Iliffe S. Alzheimer’s disease. BMJ. 2009;338:b158. 10.1136/bmj.b158 19196745

[6] Kalia LV , Lang AE. Parkinson’s disease. Lancet. 2015;386(9996):896–912. 25904081 10.1016/S0140-6736(14)61393-3

[7] Filippi M , Bar-Or A, Piehl F, et al Multiple sclerosis. Nat Rev Dis Primers. 2018;4(1):43. 30410033 10.1038/s41572-018-0041-4

[8] Lublin FD , Häring DA, Ganjgahi H, et al How patients with multiple sclerosis acquire disability. Brain J Neurol. 2022;145(9):3147–3161. 10.1093/brain/awac016 PMC9536294 35104840

[9] Hu WT , Howell JC, Ozturk T, et al CSF cytokines in aging, multiple sclerosis, and dementia. Front Immunol. 2019;10:480. 30930904 10.3389/fimmu.2019.00480 PMC6428695

[10] Li L , Mao S, Wang J, Ding X, Zen JY. Viral infection and neurological disorders—Potential role of extracellular nucleotides in neuroinflammation. ExRNA. 2019;1(1):26.

[11] Patrick KL , Bell SL, Weindel CG, Watson RO. Exploring the “multiple-hit hypothesis” of neurodegenerative disease: Bacterial infection comes up to bat. Front Cell Infect Microbiol. 2019;9:138. 31192157 10.3389/fcimb.2019.00138 PMC6546885

[12] Haahr S , Höllsberg P. Multiple sclerosis is linked to Epstein-Barr virus infection. Rev Med Virol. 2006;16(5):297–310. 16927411 10.1002/rmv.503

[13] Thacker EL , Mirzaei F, Ascherio A. Infectious mononucleosis and risk for multiple sclerosis: A meta-analysis. Ann Neurol. 2006;59(3):499–503. 16502434 10.1002/ana.20820

[14] Virtanen JO , Jacobson S. Viruses and multiple sclerosis. CNS Neurol Disord Drug Targets. 2012;11(5):528–544. 22583435 10.2174/187152712801661220 PMC4758194

[15] Fang F , Wirdefeldt K, Jacks A, Kamel F, Ye W, Chen H. CNS infections, sepsis and risk of Parkinson’s disease. Int J Epidemiol. 2012;41(4):1042–1049. 22523201 10.1093/ije/dys052 PMC3429872

[16] Sipilä PN , Heikkilä N, Lindbohm JV, et al Hospital-treated infectious diseases and the risk of dementia: A large, multicohort, observational study with a replication cohort. Lancet Infect Dis. 2021;21(11):1557–1567. 34166620 10.1016/S1473-3099(21)00144-4 PMC8592915

[17] Müller L , Di Benedetto S, Pawelec G. The immune system and its dysregulation with aging. In: Harris JR, Korolchuk VI, eds. Biochemistry and cell biology of ageing: Part II clinical science. Vol. 91. Springer Singapore; 2019:21–43. Accessed 2 June 2022. http://link.springer.com/10.1007/978-981-13-3681-2_2 10.1007/978-981-13-3681-2_2 30888648

[18] Perry VH , Nicoll JAR, Holmes C. Microglia in neurodegenerative disease. Nat Rev Neurol. 2010;6(4):193–201. 20234358 10.1038/nrneurol.2010.17

[19] Schneider JL , Rowe JH, Garcia-de-Alba C, Kim CF, Sharpe AH, Haigis MC. The aging lung: Physiology, disease, and immunity. Cell. 2021;184(8):1990–2019. 33811810 10.1016/j.cell.2021.03.005 PMC8052295

[20] Gleeson M , Walsh NP. British Association of sport and exercise sciences. The BASES expert statement on exercise, immunity, and infection. J Sports Sci. 2012;30(3):321–324. 22132765 10.1080/02640414.2011.627371

[21] Jung MH , Yi SW, An SJ, et al Association of physical activity and lower respiratory tract infection outcomes in patients with cardiovascular disease. J Am Heart Assoc. 2022;11(6):e023775. 10.1161/JAHA.121.023775 PMC9075310 35132873

[22] Scalfari A , Knappertz V, Cutter G, Goodin DS, Ashton R, Ebers GC. Mortality in patients with multiple sclerosis. Neurology. 2013;81(2):184–192. 23836941 10.1212/WNL.0b013e31829a3388 PMC3770174

[23] James BD , Leurgans SE, Hebert LE, Scherr PA, Yaffe K, Bennett DA. Contribution of Alzheimer disease to mortality in the United States. Neurology. 2014;82(12):1045–1050. 24598707 10.1212/WNL.0000000000000240 PMC3962992

[24] Xu J , Gong DD, Man CF, Fan Y. Parkinson’s disease and risk of mortality: Meta-analysis and systematic review. Acta Neurol Scand. 2014;129(2):71–79. 24256347 10.1111/ane.12201

[25] Buljevac D , Flach HZ, Hop WCJ, et al Prospective study on the relationship between infections and multiple sclerosis exacerbations. Brain. 2002;125(5):952–960. 11960885 10.1093/brain/awf098

[26] Asby D , Boche D, Allan S, Love S, Miners JS. Systemic infection exacerbates cerebrovascular dysfunction in Alzheimer’s disease. Brain J Neurol. 2021;144(6):1869–1883. 10.1093/brain/awab094 PMC8320299 33723589

[27] Lopez-Rodriguez AB , Hennessy E, Murray CL, et al Acute systemic inflammation exacerbates neuroinflammation in Alzheimer’s disease: IL-1β drives amplified responses in primed astrocytes and neuronal network dysfunction. Alzheimers Dement. 2021;17(10):1735–1755. 34080771 10.1002/alz.12341 PMC8874214

[28] Ineichen C , Baumann-Vogel H, Sitzler M, Waldvogel D, Baumann CR. Worsened Parkinson’s disease progression: Impact of the COVID-19 pandemic. J Park Dis. 2021;11(4):1579–1583. 10.3233/JPD-212779 34397421

[29] Sudlow C , Gallacher J, Allen N, et al UK Biobank: An open access resource for identifying the causes of a wide range of complex diseases of middle and old age. PLoS Med. 2015;12(3):e1001779. 10.1371/journal.pmed.1001779 PMC4380465 25826379

[30] Zagai U , Lichtenstein P, Pedersen NL, Magnusson PKE. The Swedish twin registry: Content and management as a research infrastructure. Twin Res Hum Genet. 2019;22(6):672–680. 31747977 10.1017/thg.2019.99

[31] Patientregistret. Socialstyrelsen . Accessed 8 June 2022. https://www.socialstyrelsen.se/statistik-och-data/register/patientregistret/

[32] Pearce N . Incidence density matching with a simple SAS computer program. Int J Epidemiol. 1989;18(4):981–984. 2621036 10.1093/ije/18.4.981

[33] Austin SR , Wong YN, Uzzo RG, Beck JR, Egleston BL. Why summary comorbidity measures such as the Charlson comorbidity index and elixhauser score work. Med Care. 2015;53(9):e65-72. 23703645 10.1097/MLR.0b013e318297429c PMC3818341

[34] Docherty AB , Harrison EM, Green CA, et al Features of 20133 UK patients in hospital with COVID-19 using the ISARIC WHO clinical characterisation protocol: Prospective observational cohort study. BMJ. 2020;369:m1985. 10.1136/bmj.m1985 PMC7243036 32444460

[35] Huang YT , Yang HI. Causal mediation analysis of survival outcome with multiple mediators. Epidemiology. 2017;28(3):370–378. 28296661 10.1097/EDE.0000000000000651 PMC5408128

[36] Altman DG . Statistics notes: Interaction revisited: The difference between two estimates. BMJ. 2003;326(7382):219–219. 12543843 10.1136/bmj.326.7382.219 PMC1125071

[37] Luna G , Alping P, Burman J, et al Infection risks among patients with multiple sclerosis treated with fingolimod, natalizumab, rituximab, and injectable therapies. JAMA Neurol. 2020;77(2):184–191. 31589278 10.1001/jamaneurol.2019.3365 PMC6784753

[38] Smith TE , Kister I. Infection mitigation strategies for multiple sclerosis patients on oral and monoclonal disease-modifying therapies. Curr Neurol Neurosci Rep. 2021;21(7):36. 34009478 10.1007/s11910-021-01117-y PMC8132488

[39] Williamson EM , Berger JR. Infection risk in patients on multiple sclerosis therapeutics. CNS Drugs. 2015;29(3):229–244. 25761739 10.1007/s40263-015-0226-2

[40] Castelo-Branco A , Chiesa F, Conte S, et al Infections in patients with multiple sclerosis: A national cohort study in Sweden. Mult Scler Relat Disord. 2020;45:102420. 10.1016/j.msard.2020.102420 32736217

[41] Cameron E , Rog D, McDonnell G, Overell J, Pearson O, French DP. Factors influencing multiple sclerosis disease-modifying treatment prescribing decisions in the United Kingdom: A qualitative interview study. Mult Scler Relat Disord. 2019;27:378–382. 30500689 10.1016/j.msard.2018.11.023

[42] Odoardi F , Sie C, Streyl K, et al T cells become licensed in the lung to enter the central nervous system. Nature. 2012;488(7413):675–679. 22914092 10.1038/nature11337

[43] Hosang L , Canals RC, van der Flier FJ, et al The lung microbiome regulates brain autoimmunity. Nature. 2022;603(7899):138–144. 35197636 10.1038/s41586-022-04427-4

[44] Smith KA , Hiyoshi A, Burkill S, et al Hospital diagnosed pneumonia before age 20 years and multiple sclerosis risk. BMJ Neurol Open. 2020;2(1):e000044. 10.1136/bmjno-2020-000044 PMC7903180 33681783

[45] Brunnström HR , Englund EM. Cause of death in patients with dementia disorders. Eur J Neurol. 2009;16(4):488–492. 19170740 10.1111/j.1468-1331.2008.02503.x

[46] Burns A , Jacoby R, Luthert P, Levy R. Cause of death in Alzheimer’s disease. Age Ageing. 1990;19(5):341–344. 2251969 10.1093/ageing/19.5.341

[47] Suttrup I , Warnecke T. Dysphagia in Parkinson’s disease. Dysphagia. 2016;31(1):24–32. 26590572 10.1007/s00455-015-9671-9

[48] Morgante L , Salemi G, Meneghini F, et al Parkinson Disease survival: A population-based study. Arch Neurol. 2000;57(4):507–512. 10768625 10.1001/archneur.57.4.507

[49] Pepper PV , Goldstein MK. Postoperative complications in Parkinson’s disease. J Am Geriatr Soc. 1999;47(8):967–972. 10443858 10.1111/j.1532-5415.1999.tb01292.x

[50] Mueller MC , Jüptner U, Wuellner U, et al Parkinson’s disease influences the perioperative risk profile in surgery. Langenbecks Arch Surg. 2009;394(3):511–515. 18712410 10.1007/s00423-008-0404-5

[51] Hu Y , Yang H, Hou C, et al COVID-19 related outcomes among individuals with neurodegenerative diseases: A cohort analysis in the UK biobank. BMC Neurol. 2022;22(1):15. 34996388 10.1186/s12883-021-02536-7 PMC8739517

[52] Klein RS , Garber C, Howard N. Infectious immunity in the central nervous system and brain function. Nat Immunol. 2017;18(2):132–141. 28092376 10.1038/ni.3656 PMC5815515

[53] Lutshumba J , Nikolajczyk BS, Bachstetter AD. Dysregulation of systemic immunity in aging and dementia. Front Cell Neurosci. 2021;15:652111. 10.3389/fncel.2021.652111 PMC8258160 34239415

[54] Wang H , Liu X, Tan C, et al Bacterial, viral, and fungal infection-related risk of Parkinson’s disease: Meta-analysis of cohort and case-control studies. Brain Behav. 2020;10(3):e01549. 10.1002/brb3.1549 PMC7066372 32017453

[55] Gilden DH . Infectious causes of multiple sclerosis. Lancet Neurol. 2005;4(3):195–202. 15721830 10.1016/S1474-4422(05)01017-3 PMC7129502

[56] Offner H , Vandenbark AA, Hurn PD. Effect of experimental stroke on peripheral immunity: CNS ischemia induces profound immunosuppression. Neuroscience. 2009;158(3):1098–1111. 18597949 10.1016/j.neuroscience.2008.05.033 PMC2666964

[57] Liu Q , Jin WN, Liu Y, et al Brain ischemia suppresses immunity in the periphery and brain via different neurogenic innervations. Immunity. 2017;46(3):474–487. 28314594 10.1016/j.immuni.2017.02.015

[58] Engel O , Akyüz L, da Costa Goncalves AC, et al Cholinergic pathway suppresses pulmonary innate immunity facilitating pneumonia after stroke. Stroke. 2015;46(11):3232–3240. 26451017 10.1161/STROKEAHA.115.008989

[59] Hoffmann S , Harms H, Ulm L, et al Stroke-induced immunodepression and dysphagia independently predict stroke-associated pneumonia - the PREDICT study. J Cereb Blood Flow Metab. 2017;37(12):3671–3682. 27733675 10.1177/0271678X16671964 PMC5718319

[60] Andersson U , Tracey KJ. Neural reflexes in inflammation and immunity. J Exp Med. 2012;209(6):1057–1068. 22665702 10.1084/jem.20120571 PMC3371736

[61] Perry VH , Cunningham C, Holmes C. Systemic infections and inflammation affect chronic neurodegeneration. Nat Rev Immunol. 2007;7(2):161–167. 17220915 10.1038/nri2015

[62] Jick SS , Li L, Falcone GJ, Vassilev ZP, Wallander MA. Mortality of patients with multiple sclerosis: A cohort study in UK primary care. J Neurol. 2014;261(8):1508–1517. 24838537 10.1007/s00415-014-7370-3 PMC4119255

[63] Smyrke N , Dunn N, Murley C, Mason D. Standardized mortality ratios in multiple sclerosis: Systematic review with meta-analysis. Acta Neurol Scand. 2022;145(3):360–370. 34820847 10.1111/ane.13559

[64] Huh TH , Yoon JL, Cho JJ, Kim MY, Ju YS. Survival analysis of patients with Alzheimer’s disease: A study based on data from the Korean national health insurance services’ senior cohort database. Korean J Fam Med. 2020;41(4):214–221. 32321203 10.4082/kjfm.18.0114 PMC7385296

