# Supplementary material for: Infections among individuals with multiple sclerosis, Alzheimer’s disease and Parkinson’s disease
Source: Brain Commun. 2023 Mar 16;5(2):fcad065. doi: 10.1093/braincomms/fcad065 (PMC10053639; doi:10.1093/braincomms/fcad065)
Supplement: fcad065_Supplementary_Data [file fcad065_supplementary_data.docx]

**Supplementary material**

Supplementary Table1 International Classification of Disease (ICD) codes), ninth (ICD-9) and tenth (ICD-10) revisions for Charlson comorbidity index used in this study

| **Charlson comorbidity index score** |  |  |  |
| --- | --- | --- | --- |
| 1 | Myocardial infarction | I21, I22, I25.2 | 410, 412 |
| 1 | Congestive heart failure | I11.0, I13.0, I13.2, I50 | 428 |
| 1 | Peripheral vascular disease | I70, I71, I73.1, I73.8, I73.9, I77.1, I79.0, I79.2, K55.1, K55.8, K55.9, R02, Z95.8, Z95.9 | 440, 441, 443.9, 785.4, V43.4 |
| 1 | Cerebrovascular disease | G45, G46, I60-I69 | 430-438 |
| 1 | Dementia | F00-F03, F05.1, G30, G31.1 | 290 |
| 1 | Chronic pulmonary disease | J40-J47, J60-J67, J68.4, J70, J84.1, J92.0, J96.1, J98.2 | 490-496, 500-505 |
| 1 | Connective tissue disease | M05, M06, M30, M31.5, M32-M34, M35.1, M35.3, M36.0 | 710.0, 710.1, 710.4, 714.0, 714.1, 714.2, 714.8, 714.9, 725 |
| 1 | Ulcer disease | K25-K28 | 531-534 |
| 1 | Mild liver disease | B18, K70.0-K70.3, K70.9, K71.3-K71.5, K71.7, K73, K74, K76.0, K76.2-K76.4, K76.8, K76.9 | 517.2, 517.4-517.6 |
| 1 | Diabetes mellitus | E10.0, E10.1, E10.6, E10.8, E10.9, E10.0, E11.1, E11.6, E11.8, E11.9, E12.0, E121, E12.6, E12.8, E12.9, E13.0, E13.1, E13.6, E13.8, E13.9, E14.0, E14.1, E14.6, E14.8, E14.9 | 250.0-250.2 |
| 2 | Hemiplegia | G04.1, G11.4, G80.1, G80.2, G81, G82, G83.0, G83.1, G83.2, G83.9, G83.3, G83.4 | 342, 244.1 |
| 2 | Moderate/severe renal disease | I12.0, I13.1, N03.2-N03.7, N05.2-N05.7, N18, N19, N25.0, Z94.0, Z99.2 | 582, 583.0-583.2, 583.4, 583.6, 583,7, 585, 586, 588, V420, V56 |
| 2 | Diabetes mellitus with chronic complications | E10.2-E10.5, E10.7, E11.7, E12.7, E13.7, E14.7, E11.2-E11.5, E12.2-E12.5, E13.2-E13.5, E14.2-E14.5 | 250.3-250.5 |
| 2 | Any tumor | C00-C26, C30-C34, C37-C41, C43, C45-C58, C60-C76, C97 | 140-165, 170-175, 179-195 |
| 2 | Leukemia | C91-C95 | 204-208 |
| 2 | Lymphoma | C81-C85, C88, C90, C96 | 200-203 |
| 3 | Moderate/severe liver disease | I85, K70.4, K72, K76.6 | 456.0-456.2, 572.2-572.4, 572.8 |
| 6 | Metastatic solid tumor | C77-C80 | 196-199 |
| 6 | AIDS | B20-B24 | 042-044 |

Supplementary Table 2 International Classification of Disease (ICD) codes, eighth (ICD-8), ninth (ICD-9) and tenth (ICD-10) revisions for diagnoses of infections used in this study

|  | **ICD version** | **ICD** |
| --- | --- | --- |
| *Specific site of infection* |  |  |
| Genitourinary | 8 | 016,590,595,597 |
|  | 9 | 016,590,595,597 |
|  | 10 | N30,N34,O23 |
| Respiratory | 8 | 010,011,012,033,034,075,115,116,490,501,503,510,460-466,470-474,480-486 |
|  | 9 | 010,011,012,033,034,075,115,116,473,475,487,490,510, 460-466,480-486 |
|  | 10 | A15,A16,A37,A38,B27,B39,B40,B41,B42,B44,B59,J00-J06,J10,J12-J18,J20-J22,J32,J36,J40-J42,J86,P23 |
| Other/unspecified | 8 | 000-009,013,014,015,017,018,019,020-027,030,031,032,035,036,037,038,039,040-046,050-057,060-068,070,071,072,  073,074,076,077,078,079,080-089,090-099,100-104,110,111,112,113,114,117,120-129,130-136,292,320,323,324,360,  362,363,380,381,382,383,390-392,420,421,422,540,567,610-616,620,622,630,635,670,678,680-686,710,720,732,763,  Y41 |
|  | 9 | 001-009,013,014,015,017,018,020-027,030,031,032,035,036,037,038,039,040,041,045-049,050-057,060-066,070,071,  072,073,074,076,077,078,079,080-088,090-099,100-104,110,111,112,113,114,117,118,120,121-129,130-136,137-139,  320,323,326,370,372,381,382,383,390-392,420,421,422,540,567,614-616,647,670,675,680-686,711,730,771 |
|  | 10 | A00-A09,A17,A18,A19,A20-A28,A30-A36,A39-A49,A50-A60,A63,A64,A65-A69,A70-A74,A75-A79,A80-A89,A90-A99,B00-B09,B15-B19,B20-B24,B25,B26,B28-B34,B35,B36,B37,B38,B43,B45,B46,B47,B48,B49,B50-B58,B60-B64,B65-B83,B85-B89,B90-B94,B95,B96,B97,B99,G00-G05,H10,H16,H32,H60,H70,I00,I01,I02,I30,I33,I40,K35,  K65,K67,L00-L08,M00,M01,M02,M03,M86,N70-N77,O85,O86,O91,O98,P35-P39,V02,Z21,Z22 |
| *Type of infection* |  |  |
| Bacterial | 8 | 595,597,612,613,614,616,620,622,630,635,670,678,680,000,001,002,003,004,005,073,076,080,081,082,083,320,362,  380,381,382,383,421,461,481,482,501,510,567,590,681,682,684,710,720,010-019,020-027,030-039,090-099,100-104,390-392 |
|  | 9 | 001,002,003,004,005,073,076,077,078,079,080,081,082,083,320,381,382,383,383,421,461,475,481,482,510,567,590,595,597,670,730,010-018,020-027,030-041,090-099,100-104,390-392,614-616,680-686 |
|  | 10 | A00,A01,A02,A03,A04,A05,A15-A19,A20-A28,A30-A49,A50-A58,A65-A69,A70-A74,A75-A79,B95,B96,  G00,G01,H60,H70,I00-I02,I33,J01,J13,J14,J15,J36,J86,K65,L00-L08,M00,M86,N30,N34,N70-N77,O23,O85,O86,  P36 |
| Viral | 8 | 075,360,420,422,460,464,465,466,480,040-046,050-057,060-068,070-079,470-474 |
|  | 9 | 070,071,072,074,075,077,078,079,372,420,422,460,464,465,466,480,487,647,711,045-049,050-057,060-066 |
|  | 10 | A08,A60,A80-A89,A90-A99,B00-B09,B15-B19,B20-B24,B25-B34,B27,B97,B99,H10,I30,I40,J00,J04, J05,J06,J10,J12,J20,J21,O98,P35,Z21 |
| Other | 8 | 006,007,008,009,084,085,086,087,088,089,110-117,120-129,130-136,292,323,324,363,462,463,483,484,485,486,  490,503,540,610,611,615,683,685,686,732,763,Y41 |
|  | 9 | 006,007,008,009,084,085,086,087,088,110-118,120-129, 130-136,137-139,323,326,370,462,463,473,483,484,485,  486,490,540,675,771 |
|  | 10 | A06,A07,A09,A59,A63,A64,B35-B49,B50-B64,B65-B83,B85-B89,B90-B94,G02,G04,G05,H16,H32,J02,J03,J16,  J17,J18,J22,J32,J40,J41,J42,K35,K67,M01,M02,M03,O91,P23,P37,P38,P39,V02,Z22 |
| *Specific infection* |  |  |
| Pneumonia | 8 | 480-486 |
|  | 9 | 480-486 |
|  | 10 | J12-J18, P23 |

Supplementary Table 3 Characteristics of individuals included in the cohort studies of multiple sclerosis (MS), Alzheimer’s disease (AD) and Parkinson’s disease (PD), based on the UK biobank and the Swedish Twin Registry

|  | UK Biobank | | | | | | Swedish Twin Registry | | | | | |
| --- | --- | --- | --- | --- | --- | --- | --- | --- | --- | --- | --- | --- |
| Characteristics | MS patients  (n= 2,023) | Matched controls  (n= 10,115) | AD patients  (n= 2,200) | Matched controls  (n=10,999) | PD patients  (n=3,050) | Matched controls  (n=15,249) | MS patients  (n=230) | Co-twin controls  (n=230) | AD patients  (n=885) | Co-twin controls  (n=899) | PD patients  (n=626) | Co-twin controls  (n=626) |
| Number of controls that developed MS/AD/PD during follow-up, n. (%) |  | 19 (0.2) |  | 75 (0.7) |  | 83 (0.5) |  | 8 (3.4) |  | 112 (12.5) |  | 13 (2.1) |
| Age, mean (SD) | 54.7 (10.1) | 54.7 (10.1) | 73.2 (5.1) | 73.2 (5.1) | 69.1 (7.2) | 69.1 (7.2) | 47.9 (15.0) | 47.9 (15.0) | 77.2 (7.2) | 77.2 (7.2) | 71.5 (10.0) | 71.5 (10.0) |
| Sex, n. (%) |  |  |  |  |  |  |  |  |  |  |  |  |
| Male | 567 (28.0) | 2835 (28.0) | 1067 (48.5) | 5335 (48.5) | 1877 (61.5) | 9385 (61.5) | 61 (26.5) | 88 (38.3) | 306 (34.6) | 336 (37.4) | 321 (51.3) | 314 (50.2) |
| Female | 1456 (72.0) | 7280 (72.0) | 1133 (51.5) | 5664 (51.5) | 1173 (38.5) | 5864 (38.5) | 169 (73.5) | 142 (61.7) | 579 (65.4) | 563 (62.6) | 305 (48.7) | 312 (49.8) |
| Zygotic, n. (%) |  |  |  |  |  |  |  |  |  |  |  |  |
| Monozygotic |  |  |  |  |  |  | 70 (30.4) | 70 (30.4) | 253 (28.6) | 260 (28.9) | 173 (27.6) | 173 (27.6) |
| Dizygotic same sex |  |  |  |  |  |  | 93 (40.4) | 93 (40.4) | 433 (48.9) | 440 (48.9) | 292 (46.6) | 292 (46.6) |
| Dizygotic opposite sex |  |  |  |  |  |  | 61 (26.5) | 61 (26.5) | 158 (17.9) | 158 (17.6) | 125 (20.0) | 125 (20.0) |
| Unknown |  |  |  |  |  |  | 6 (2.6) | 6 (2.6) | 41 (4.6) | 41 (4.6) | 36 (5.8) | 36 (5.8) |
| Educational attainment (Sweden) |  |  |  |  |  |  |  |  |  |  |  |  |
| Compulsory school or elementary school |  |  |  |  |  |  | 41 (17.8) | 38 (16.5) | 356 (40.2) | 384 (42.7) | 217 (34.7) | 220 (35.1) |
| Upper secondary education |  |  |  |  |  |  | 70 (30.4) | 93 (40.4) | 206 (23.3) | 231 (25.7) | 151 (24.1) | 164 (26.2) |
| University |  |  |  |  |  |  | 65 (28.3) | 63 (27.4) | 88 (9.9) | 93 (10.3) | 85 (13.6) | 91 (14.5) |
| Others |  |  |  |  |  |  | 2 (0.9) | 0 (0.0) | 35 (4.0) | 22 (2.4) | 22 (3.5) | 17 (2.7) |
| Unknown |  |  |  |  |  |  | 52 (22.6) | 36 (15.7) | 200 (22.6) | 169 (18.8) | 151 (24.1) | 134 (21.4) |
| Townsend deprivation index, mean (SD) | -1.2 (3.1) | -1.2 (3.1) | -1.1 (3.2) | -1.4 (3.0) | -1.4 (3.1) | -1.4 (3.1) |  |  |  |  |  |  |
| Educational attainment (UK) |  |  |  |  |  |  |  |  |  |  |  |  |
| College or University degree | 642 (31.7) | 3168 (31.3) | 408 (18.5) | 2632 (23.9) | 827 (27.1) | 4051 (26.6) |  |  |  |  |  |  |
| A levels/AS levels or equivalent | 264 (13.0) | 1134 (11.2) | 174 (7.9) | 913 (8.3) | 267 (8.8) | 1373 (9.0) |  |  |  |  |  |  |
| O levels/GCSEs or equivalent | 459 (22.7) | 2296 (22.7) | 403 (18.3) | 2183 (19.8) | 587 (19.2) | 2881 (18.9) |  |  |  |  |  |  |
| CSEs or equivalent | 119 (5.9) | 610 (6.0) | 48 (2.2) | 281 (2.6) | 87 (2.9) | 427 (2.8) |  |  |  |  |  |  |
| NVQ or HND or HNC or equivalent | 89 (4.4) | 642 (6.3) | 174 (7.9) | 783 (7.1) | 226 (7.4) | 1212 (7.9) |  |  |  |  |  |  |
| Other professional qualifications | 102 (5.0) | 518 (5.1) | 128 (5.8) | 735 (6.7) | 186 (6.1) | 966 (6.3) |  |  |  |  |  |  |
| Unknown | 348 (17.2) | 1747 (17.3) | 865 (39.3) | 3472 (31.6) | 870 (28.5) | 4339 (28.5) |  |  |  |  |  |  |
| Annual household income, £ |  |  |  |  |  |  |  |  |  |  |  |  |
| <18,000 | 516 (25.5) | 2007 (19.8) | 746 (33.9) | 3199 (29.1) | 888 (29.1) | 3951 (25.9) |  |  |  |  |  |  |
| 18,000-30,999 | 441 (21.8) | 2094 (20.7) | 517 (23.5) | 2805 (25.5) | 716 (23.5) | 3750 (24.6) |  |  |  |  |  |  |
| 31,000-51,999 | 398 (19.7) | 2198 (21.7) | 211 (9.6) | 1759 (16.0) | 492 (16.1) | 2805 (18.4) |  |  |  |  |  |  |
| 52,000-100,000 | 260 (12.9) | 1819 (18.0) | 92 (4.2) | 777 (7.1) | 248 (8.1) | 1588 (10.4) |  |  |  |  |  |  |
| >100,000 | 54 (2.7) | 405 (4.0) | 23 (1.0) | 194 (1.8) | 77 (2.5) | 383 (2.5) |  |  |  |  |  |  |
| Unknown | 354 (17.5) | 1592 (15.7) | 611 (27.8) | 2265 (20.6) | 629 (20.6) | 2772 (18.2) |  |  |  |  |  |  |
| Charlson comorbidity index, n. (%) |  |  |  |  |  |  |  |  |  |  |  |  |
| 0 | 1550 (76.6) | 7750 (76.6) | 854 (38.8) | 4270 (38.8) | 1647 (54.0) | 8235 (54.0) |  |  |  |  |  |  |
| 1 | 229 (11.3) | 1145 (11.3) | 465 (21.1) | 2324 (21.1) | 551 (18.1) | 2755 (18.1) |  |  |  |  |  |  |
| ≥2 | 244 (12.1) | 1220 (12.1) | 881 (40.0) | 4405 (40.0) | 852 (27.9) | 4259 (27.9) |  |  |  |  |  |  |

Supplementary Table 4 Associations of multiple sclerosis (MS), Alzheimer’s disease (AD), and Parkinson’s disease (PD) with the risk of inpatient infections, a matched cohort analysis in UK Biobank

|  | MS | |  | AD | |  | PD | |  |
| --- | --- | --- | --- | --- | --- | --- | --- | --- | --- |
|  | Exposed  (cases/person-years, IR) | Unexposed  (cases/person-years, IR) | HR (95% CI) | Exposed  (cases/person-years, IR) | Unexposed  (cases/person-years, IR) | HR (95% CI) | Exposed  (cases/person-years, IR) | Unexposed  (cases/person-years, IR) | HR (95% CI) |
| Any infection | 763/18681 (40.84) | 1817/108898 (16.69) | 2.45 (2.24-2.69) | 848/3808 (222.69) | 1411/30876 (45.70) | 5.06 (4.58-5.59) | 1224/11033 (110.94) | 2425/74172 (32.69) | 3.72 (3.44-4.01) |
| **Site of infection** |  |  |  |  |  |  |  |  |  |
| Genitourinary | 50/22894 (2.18) | 69/119141 (0.58) | 3.78 (2.56-5.60) | 5/5000 (1.00) | 50/33272 (1.50) | 0.66 (0.24-1.77) | 30/14074 (2.13) | 96/80543 (1.19) | 1.89 (1.23-2.91) |
| Respiratory | 375/21656 (17.32) | 747/116003 (6.44) | 2.89 (2.53-3.30) | 520/4389 (118.48) | 703/32246 (21.80) | 5.34 (4.69-6.09) | 706/12787 (55.21) | 1239/78070 (15.87) | 3.91 (3.53-4.33) |
| Other/unspecified | 633/19662 (32.19) | 1399/111501 (12.55) | 2.55 (2.31-2.82) | 574/4186 (137.12) | 1016/31673 (32.08) | 4.31 (3.84-4.85) | 923/11813 (78.13) | 1700/76204 (22.31) | 3.83 (3.51-4.19) |
| **Type of infection** |  |  |  |  |  |  |  |  |  |
| Bacterial | 523/20141 (25.97) | 1008/113519 (8.88) | 2.94 (2.63-3.29) | 443/4413 (100.39) | 703/32243 (21.80) | 4.75 (4.15-5.44) | 751/12275 (61.18) | 1279/77407 (16.52) | 4.06 (3.67-4.48) |
| Viral | 122/22642 (5.39) | 263/118174 (2.23) | 2.32 (1.86-2.91) | 92/4897 (18.79) | 175/33101 (5.29) | 3.48 (2.61-4.64) | 132/13889 (9.50) | 314/80052 (3.92) | 2.57 (2.07-3.19) |
| Other | 481/21204 (22.68) | 1077/114412 (9.41) | 2.48 (2.21-2.78) | 608/4223 (143.97) | 943/31775 (29.68) | 4.80 (4.27-5.40) | 861/12306 (69.97) | 1608/77005 (20.88) | 3.74 (3.41-4.10) |
| **Specific infection** |  |  |  |  |  |  |  |  |  |
| Pneumonia | 257/22427 (11.46) | 407/118121 (3.45) | 3.53 (2.99-4.18) | 367/4640 (79.09) | 445/32739 (13.59) | 5.75 (4.91-6.73) | 493/13399 (36.79) | 784/79386 (9.88) | 4.26 (3.76-4.82) |

*Conditioned on pair matched with age, sex, and Charlson comorbidity index on index date, and additionally adjusted for annual household income, educational attainment, and Townsend deprivation index.

IR: incidence rate per 1000 person-years; HR: hazard ratio; CI: confidence interval

Supplementary Table 5 Associations of multiple sclerosis (MS), Alzheimer’s disease (AD), and Parkinson’s disease (PD) with the risk of hospital treated infections, a within-twin pair analysis in Swedish Twin Registry

|  | MS | |  | AD | |  | PD | |  |
| --- | --- | --- | --- | --- | --- | --- | --- | --- | --- |
|  | Exposed  (cases/person-years, IR) | Unexposed  (cases/person-years, IR) | HR (95% CI) | Exposed  (cases/person-years, IR) | Unexposed  (cases/person-years, IR) | HR (95% CI) | Exposed  (cases/person-years, IR) | Unexposed  (cases/person-years, IR) | HR (95% CI) |
| Any infection | 107/2010 (53.23) | 73/2518 (28.99) | 1.78 (1.21-2.62) | 240/3258 (73.66) | 286/4619 (61.92) | 1.50 (1.19-1.88) | 266/2967 (89.65) | 208/4569 (45.52) | 2.30 (1.79-2.95) |
| **Site of infection** |  |  |  |  |  |  |  |  |  |
| Genitourinary | 28/2486 (11.26) | 9/2966 (3.03) | 3.97 (1.25-12.62) | 34/3693 (9.21) | 29/5435 (5.34) | 2.70 (1.22-6.01) | 41/3665 (11.19) | 33/5333 (6.19) | 2.29 (1.15-4.57) |
| Respiratory | 53/2450 (21.63) | 24/2890 (8.30) | 3.59 (1.80-7.14) | 129/3586 (35.97) | 142/5173 (27.45) | 1.73 (1.24-2.40) | 150/3486 (43.03) | 103/5100 (20.20) | 2.76 (1.94-3.93) |
| Other/unspecified | 83/2170 (38.25) | 58/2606 (22.26) | 1.58 (1.04-2.41) | 128/3506 (36.51) | 188/4922 (38.20) | 1.27 (0.95-1.71) | 166/3237 (51.28) | 139/4876 (28.51) | 2.12 (1.57-2.87) |
| **Type of infection** |  |  |  |  |  |  |  |  |  |
| Bacterial | 82/2175 (37.70) | 47/2684 (17.51) | 1.71 (1.11-2.65) | 137/3492 (39.23) | 166/5092 (32.60) | 1.45 (1.07-1.97) | 168/3280 (51.22) | 135/4962 (27.21) | 2.14 (1.58-2.89) |
| Viral | 32/2518 (12.71) | 23/2889 (7.96) | 1.92 (0.95-3.87) | 69/3647 (18.92) | 97/5146 (18.85) | 1.14 (0.76-1.70) | 59/3637 (16.22) | 61/5193 (11.75) | 2.69 (1.55-4.65) |
| Other | 65/2382 (27.29) | 34/2801 (12.14) | 2.42 (1.39-4.21) | 107/3614 (29.61) | 131/5209 (25.15) | 1.53 (1.07-2.18) | 142/3432 (41.38) | 104/5113 (20.34) | 2.72 (1.91-3.88) |
| **Specific infection** |  |  |  |  |  |  |  |  |  |
| Pneumonia | 40/2539 (15.75) | 14/2950 (4.75) | 6.24 (2.18-17.84) | 106/3658 (28.98) | 114/5290 (21.55) | 1.95 (1.32-2.86) | 138/3543 (38.95) | 82/5240 (15.65) | 3.24 (2.19-4.80) |

*Conditioned on twin pair and additionally adjusted for sex and education attainment.

IR: incidence rate per 1000 person-years; HR: hazard ratio; CI: confidence interval

Supplementary Table 6 Associations of multiple sclerosis (MS), Alzheimer’s disease (AD), and Parkinson’s disease (PD) with the risk of inpatient infection within five years before the diagnosis, a matched analysis in UK Biobank

|  | MS | |  | AD | |  | PD | |  |  |
| --- | --- | --- | --- | --- | --- | --- | --- | --- | --- | --- |
|  | Exposed | Unexposed | OR (95% CI)* | Exposed | Unexposed | OR (95% CI)* | Exposed | Unexposed | OR (95% CI)* |  |
| Any infection | 239/2023 (11.8) | 576/10115 (5.7) | 2.43 (2.04-2.88) | 770/2200 (35.0) | 1534/10999 (13.9) | 3.48 (3.12-3.89) | 607/3050 (19.9) | 1640/15249 (10.8) | 2.20 (1.98-2.46) |  |
| **Site of infection** |  |  |  |  |  |  |  |  |  |  |
| Genitourinary | 13/2023 (0.6) | 24/10115 (0.2) | 2.44 (1.14-5.20) | 18/2200 (0.8) | 83/10999 (0.8) | 1.06 (0.63-1.79) | 21/3050 (0.7) | 84/15249 (0.6) | 1.31 (0.80-2.15) |  |
| Respiratory | 88/2023 (4.3) | 211/10115 (2.1) | 2.40 (1.83-3.15) | 389/2200 (17.7) | 656/10999 (6.0) | 3.41 (2.95-3.93) | 271/3050 (8.9) | 694/15249 (4.6) | 2.13 (1.83-2.48) |  |
| Other/unspecified | 175/2023 (8.7) | 393/10115 (3.9) | 2.48 (2.04-3.01) | 524/2200 (23.8) | 1081/10999 (9.8) | 2.89 (2.56-3.27) | 439/3050 (14.4) | 1109/15249 (7.3) | 2.22 (1.96-2.51) |  |
| **Type of infection** |  |  |  |  |  |  |  |  |  |  |
| Bacterial | 117/2023 (5.8) | 288/10115 (2.8) | 2.22 (1.76-2.80) | 396/2200 (18.0) | 767/10999 (7.0) | 2.92 (2.54-3.36) | 341/3050 (11.2) | 817/15249 (5.4) | 2.30 (2.00-2.64) |  |
| Viral | 38/2023 (1.9) | 91/10115 (0.9) | 2.20 (1.46-3.31) | 81/2200 (3.7) | 180/10999 (1.6) | 2.20 (1.67-2.90) | 76/3050 (2.5) | 182/15249 (1.2) | 2.08 (1.57-2.75) |  |
| Other | 127/2023 (6.3) | 271/10115 (2.7) | 2.69 (2.13-3.39) | 497/2200 (22.6) | 932/10999 (8.5) | 3.17 (2.79-3.60) | 353/3050 (11.6) | 959/15249 (6.3) | 2.01 (1.76-2.30) |  |
| **Specific infection** |  |  |  |  |  |  |  |  |  |  |
| Pneumonia | 48/2023 (2.4) | 77/10115 (0.8) | 3.56 (2.39-5.32) | 243/2200 (11.0) | 372/10999 (3.4) | 3.49 (2.92-4.16) | 164/3050 (5.4) | 344/15249 (2.3) | 2.52 (2.07-3.08) |  |

* Conditioned on pair matched with age, sex, and Charlson comorbidity index, and additionally adjusted for annual household income, educational attainment, and Townsend deprivation index

CI: confidence interval; OR: odds ratio

Supplementary Table 7 Associations of multiple sclerosis (MS), Alzheimer’s disease (AD), and Parkinson’s disease (PD) with the risk of hospital treated infection within five years before the diagnosis, a within-twin pair analysis

|  | MS | |  | AD | |  | PD | |  |  |
| --- | --- | --- | --- | --- | --- | --- | --- | --- | --- | --- |
|  | Exposed | Unexposed | OR (95% CI)* | Exposed | Unexposed | OR (95% CI)* | Exposed | Unexposed | OR (95% CI)* |  |
| Any infection | 37/230 (16.1) | 23/230 (10.0) | 1.99 (0.95-4.18) | 195/885 (22.0) | 140/899 (15.6) | 1.60 (1.23-2.08) | 117/626 (18.7) | 68/626 (10.9) | 2.09 (1.47-2.99) |  |
| **Site of infection** |  |  |  |  |  |  |  |  |  |  |
| Genitourinary | 6/230 (2.6) | 1/230 (0.4) | Inf (0.00-Inf) | 37/885 (4.2) | 23/899 (2.6) | 1.79 (0.97-3.30) | 16/626 (2.6) | 7/626 (1.1) | 1.95 (0.71-5.38) |  |
| Respiratory | 17/230 (7.4) | 9/230 (3.9) | 2.20 (0.68-7.11) | 80/885 (9.0) | 56/899 (6.2) | 1.42 (0.98-2.06) | 51/626 (8.1) | 26/626 (4.2) | 2.24 (1.34-3.75) |  |
| Other/unspecified | 26/230 (11.3) | 16/230 (7.0) | 1.74 (0.79-3.82) | 116/885 (13.1) | 89/899 (9.9) | 1.44 (1.04-1.99) | 71/626 (11.3) | 42/626 (6.7) | 1.91 (1.24-2.95) |  |
| **Type of infection** |  |  |  |  |  |  |  |  |  |  |
| Bacterial | 19/230 (8.3) | 16/230 (7.0) | 1.21 (0.50-2.94) | 119/885 (13.4) | 86/899 (9.6) | 1.63 (1.16-2.29) | 67/626 (10.7) | 44/626 (7.0) | 1.68 (1.06-2.66) |  |
| Viral | 8/230 (3.5) | 5/230 (2.2) | 5.37 (0.60-47.86) | 37/885 (4.2) | 42/899 (4.7) | 0.84 (0.52-1.36) | 31/626 (5.0) | 15/626 (2.4) | 2.29 (1.14-4.63) |  |
| Other | 19/230 (8.3) | 7/230 (3.0) | 1.97 (0.66-5.95) | 80/885 (9.0) | 45/899 (5.0) | 1.84 (1.22-2.75) | 46/626 (7.3) | 24/626 (3.8) | 2.35 (1.32-4.19) |  |
| **Specific infection** |  |  |  |  |  |  |  |  |  |  |
| Pneumonia | 10/230 (4.3) | 5/230 (2.2) | 1.00 (0.20-4.95) | 59/885 (6.7) | 32/899 (3.6) | 1.92 (1.18-3.11) | 40/626 (6.4) | 17/626 (2.7) | 2.84 (1.48-5.43) |  |

*Conditioned on twin pair and additionally adjusted for sex and education attainment.

CI: confidence interval; OR: odds ratio
